# HIF2α contributes to antiestrogen resistance via positive bilateral crosstalk with EGFR in breast cancer cells

**DOI:** 10.18632/oncotarget.7167

**Published:** 2016-02-03

**Authors:** Muhammad Wasi Alam, Camilla Ulrika Persson, Susann Reinbothe, Julhash U. Kazi, Lars Rönnstrand, Caroline Wigerup, Henrik Jorn Ditzel, Anne E. Lykkesfeldt, Sven Påhlman, Annika Jögi

**Affiliations:** ^1^ Department of Laboratory Medicine, Translational Cancer Research, Lund University Cancer Center at Medicon Village, Lund University, Sweden; ^2^ Department of Cancer and Inflammation Research, University of Southern Denmark, and Department of Oncology, Odense University Hospital, Odense, Denmark; ^3^ Breast Cancer Group, Cell Death and Metabolism, Danish Cancer Society Research Center, Copenhagen, Denmark

**Keywords:** breast cancer, antiestrogen, endocrine therapy, hypoxia, hypoxia inducible factor

## Abstract

The majority of breast cancers express estrogen receptor α (ERα), and most patients with ERα-positive breast cancer benefit from antiestrogen therapy. The ERα-modulator tamoxifen and ERα-downregulator fulvestrant are commonly employed antiestrogens. Antiestrogen resistance remains a clinical challenge, with few effective treatments available for patients with antiestrogen-resistant breast cancer. Hypoxia, which is intrinsic to most tumors, promotes aggressive disease, with the hypoxia-inducible transcription factors HIF1 and HIF2 regulating cellular responses to hypoxia. Here, we show that the ERα-expressing breast cancer cells MCF-7, CAMA-1, and T47D are less sensitive to antiestrogens when hypoxic. Furthermore, protein and mRNA levels of HIF2α/*HIF2A* were increased in a panel of antiestrogen-resistant cells, and antiestrogen-exposure further increased HIF2α expression. Ectopic expression of HIF2α in MCF-7 cells significantly decreased sensitivity to antiestrogens, further implicating HIF2α in antiestrogen resistance. EGFR is known to contribute to antiestrogen resistance: we further show that HIF2α drives hypoxic induction of EGFR and that EGFR induces HIF2α expression. Downregulation or inhibition of EGFR led to decreased HIF2α levels. This positive and bilateral HIF2-EGFR regulatory crosstalk promotes antiestrogen resistance and, where intrinsic hypoxic resistance exists, therapy itself may exacerbate the problem. Finally, inhibition of HIFs by FM19G11 restores antiestrogen sensitivity in resistant cells. Targeting HIF2 may be useful for counteracting antiestrogen resistance in the clinic.

## INTRODUCTION

Selective estrogen receptor modulators (SERMs) like tamoxifen, and selective estrogen receptor down-regulators (SERDs) like fulvestrant, are widely used to treat estrogen receptor α (ERα)-positive breast cancer. These drugs reduce tumor growth and metastasis by counteracting ERα-induced proliferation [1]. Antiestrogen resistance frequently occurs, particularly in patients with high-stage disease [2], posing a prominent clinical difficulty. Resistance can be intrinsic (*de novo* resistance), but more commonly it arises during treatment (acquired resistance).

ERα (encoded by *ESR1* or *NR3A1*) binds estrogen (the most potent estrogen is estradiol, E2) in a hydrophobic pocket, inducing receptor conformational changes, dimer formation and transcriptional regulation of genes with ERα binding-sites in their promoters. Tamoxifen and its active metabolite 4-hydroxytamoxifen, competitively binds to the same hydrophobic pocket in ERα as E2 does, but induces a different conformational change to modulate, although not completely abolish, ERα signaling [3]. SERDs also bind to this hydrophobic pocket to induce conformational changes that abolish ERα signaling and cause proteasomal degradation of ERα [4].

Most solid tumors harbor poorly oxygenated, hypoxic, areas due to insufficient circulation. Hypoxia results in the stabilization and activation of the hypoxia-inducible transcription factor alpha-subunits HIF1α and HIF2α [5, 6]. To induce transcription, the alpha-subunits dimerize with HIFβ/ARNT and translocate to the nucleus, where HIFα/HIFβ-complexes bind hypoxia-responsive element (HRE) sites in the promoters of genes including vascular endothelial growth factor (*VEGF*) and *EPO*. Stabilization of HIF1α and HIF2α contributes to hypoxic cell survival and neovascularization [7]. It is well known that tumor hypoxia is associated with poor prognosis and therapeutic resistance [8]. Our laboratory previously reported that high HIF2α protein levels are associated with distant metastases and poor outcomes in breast cancer patients [9].

Activation of alternative growth and survival signaling pathways, e.g. human epidermal growth factor receptor (HER1 or EGFR) and HER2, has been described in antiestrogen therapy-resistant breast cancer [10, 11]. HIF1α and HIF2α are implicated in therapeutic resistance in breast cancer [12, 13], and HIF2α influences EGFR translation [14, 15] at hypoxia. To test if hypoxia *per se* can induce antiestrogen resistance and to establish the mechanisms for the potential hypoxia-induced resistance, we investigated how hypoxia and HIFs affect sensitivity to tamoxifen and fulvestrant. We observed that hypoxic conditions increased the proportion of viable cells after antiestrogen treatment. HIF2α expression was increased in antiestrogen-resistant cells, and co-treatment with the HIF-inhibitor FM19G11 restored their antiestrogen sensitivity. Ectopic expression of HIF2α significantly increased the viability of MCF-7 cells after exposure to tamoxifen or fulvestrant, further strengthening the link between HIF2α and antiestrogen resistance. EGFR expression was increased in antiestrogen-resistant cells (as previously reported for fulvestrant-resistant cells [16]) and further induced by hypoxia. Silencing HIF2α significantly lowered EGFR expression, whereas HIF2α overexpression induced EGFR. Finally, EGFR induced HIF2α expression, suggesting that these two proteins form a positive regulatory-loop that promotes antiestrogen resistance.

## RESULTS

### Effects of hypoxia on antiestrogen treatment in ERα-positive breast cancer cells

**Figure 1:**
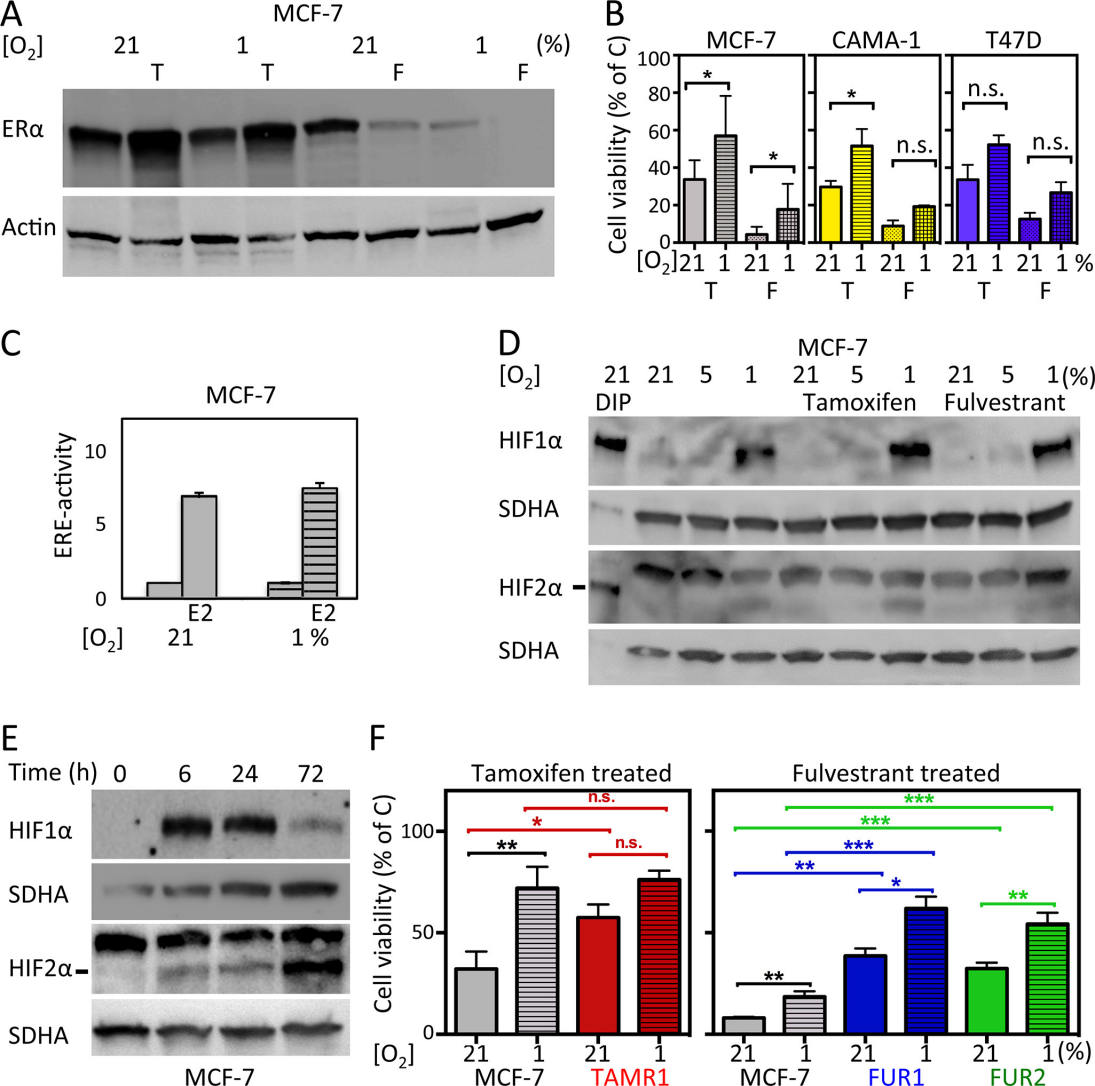
Effects of hypoxia and antiestrogen treatment in estrogen receptor-positive breast cancer cells (**A**) Treatment of MCF-7 cells with 0.5 μM tamoxifen for 72 h at normoxic and hypoxic conditions results in increased protein levels of ERα. Fulvestrant has the opposite effect. Actin was used as a loading control. (**B**) Cell viability displayed as percentage of untreated control cells (C) for three ERα-positive cell lines: MCF-7, CAMA-1, and T47D. The cells were counted after exposure to antiestrogens under hypoxic (1%) or control (21%) conditions for six days. Three independent experiments in triplicate were performed for each cell line. The differences in percentages of surviving cells were significant where indicated (*). In the other settings (n.s.), the differences were statistically significant in two of the three experiments. Student's *t*-test, significance *p < 0.05. (**C**) Transcriptional activity of ERα in MCF-7 cells analyzed by an ERE-luciferase assay under control (21%) and hypoxic (1%) conditions with and without addition of 17-β-estradiol (E2) for 24 h to the culture medium. (**D**) western blot analyses for HIF1α and HIF2α in MCF-7 cells cultured under the indicated oxygen conditions for 72 h. Dipyridyl (DIP) treatment leads to HIF α-subunit accumulation and was used to generate positive controls for western blots since the HIF2α antibody also detects a non-specific product. DIP indicates exposure for 24 h [100 μM]; less amount of sample was loaded to avoid overflow into adjacent wells. SDHA was used as a loading control. The HIF2α protein is indicated with a line. (**E**) Western blot for HIF1α and HIF2α at the indicated time points of exposure to hypoxic conditions (1% oxygen). SDHA was used as a loading control. The HIF2α protein is indicated with a line. (**F**) Cell viability (% of non-drug-treated control cells) after six days of tamoxifen [0.5 μM] or fulvestrant [0.5 μM] exposure to tamoxifen- (TAMR1) and fulvestrant- (FUR1 and 2) resistant MCF-7 cells at 21% oxygen and 1% oxygen, respectively. Data presented are the mean from three independent experiments in triplicate. Statistical analysis with Student's *t*-test, *p < 0.05, **p < 0.01, ***p < 0.001.

We hypothesized that hypoxia would reduce the effect of antiestrogen treatment, since ERα is downregulated in response to hypoxia (Figure 1A). Tamoxifen treatment resulted in increased protein expression of ERα, whereas fulvestrant treatment led to decreased protein expression of ERα (Figure 1A), as anticipated [4], and the hypoxic ERα-downregulating effect persisted in antiestrogen-treated cells (Figure 1A).

We next examined if antiestrogen sensitivity was affected by hypoxia in ERα-positive cell lines: MCF-7, CAMA-1, and T47D. All three cell lines were less sensitive to antiestrogens under hypoxic conditions (Figure 1B). However, the transcriptional activity of ERα was not affected by hypoxia as assessed by an ERα luciferase reporter assay (Figure 1C), suggesting that ERα itself is unlikely to be responsible for the decreased antiestrogen effect during hypoxia.

Since HIFs are important mediators of hypoxic adaptation, HIF1α and HIF2α protein levels were assessed in MCF-7 cells after 72 h (a time-point at which neither tamoxifen nor fulvestrant had caused significant differences in cell density) in the absence or presence of antiestrogen showing similar accumulation of both factors under hypoxic conditions (Figure 1D). Dipyridyl (DIP) treatment leads to HIFα protein accumulation by inhibiting VHL-dependent proteasomal degradation and was used as a positive control for HIF1α and HIF2α protein detection (Figure 1D). The kinetics of HIF1α and HIF2α accumulation in response to hypoxia varied, with HIF1α expression increasing prior to 6 h and declining at 72 h (Figure 1E). In contrast, HIF2α protein expression continued to increase even at 72 h of hypoxia (Figure 1E). We did not detect significant differences in cell density between control and drug-exposed cells as early as at 72 h of exposure (data not shown), which may indicate that any HIF-dependent influence on sensitivity is likely to be via the action of HIF2α as this is the dominating isoform at later time-points.

To further analyze the nature of hypoxia-induced antiestrogen resistance, we utilized a panel of antiestrogen-resistant cell lines that were generated from MCF-7 cells surviving longterm treatment with growth arresting concentration of tamoxifen (TAMR1) or fulvestrant (FUR1 and FUR2) [17–19]. As anticipated, an increased percentage of drug-resistant cells survived exposure to antiestrogens compared to parental MCF-7 cells (Figure 1F and Supplementary Figure S1). Notably, resistance was further increased under hypoxic conditions (Figure 1F and Supplementary Figure S1).

### Breast cancer cells with acquired antiestrogen resistance have increased protein levels of HIF2α, but not HIF1α

**Figure 2:**
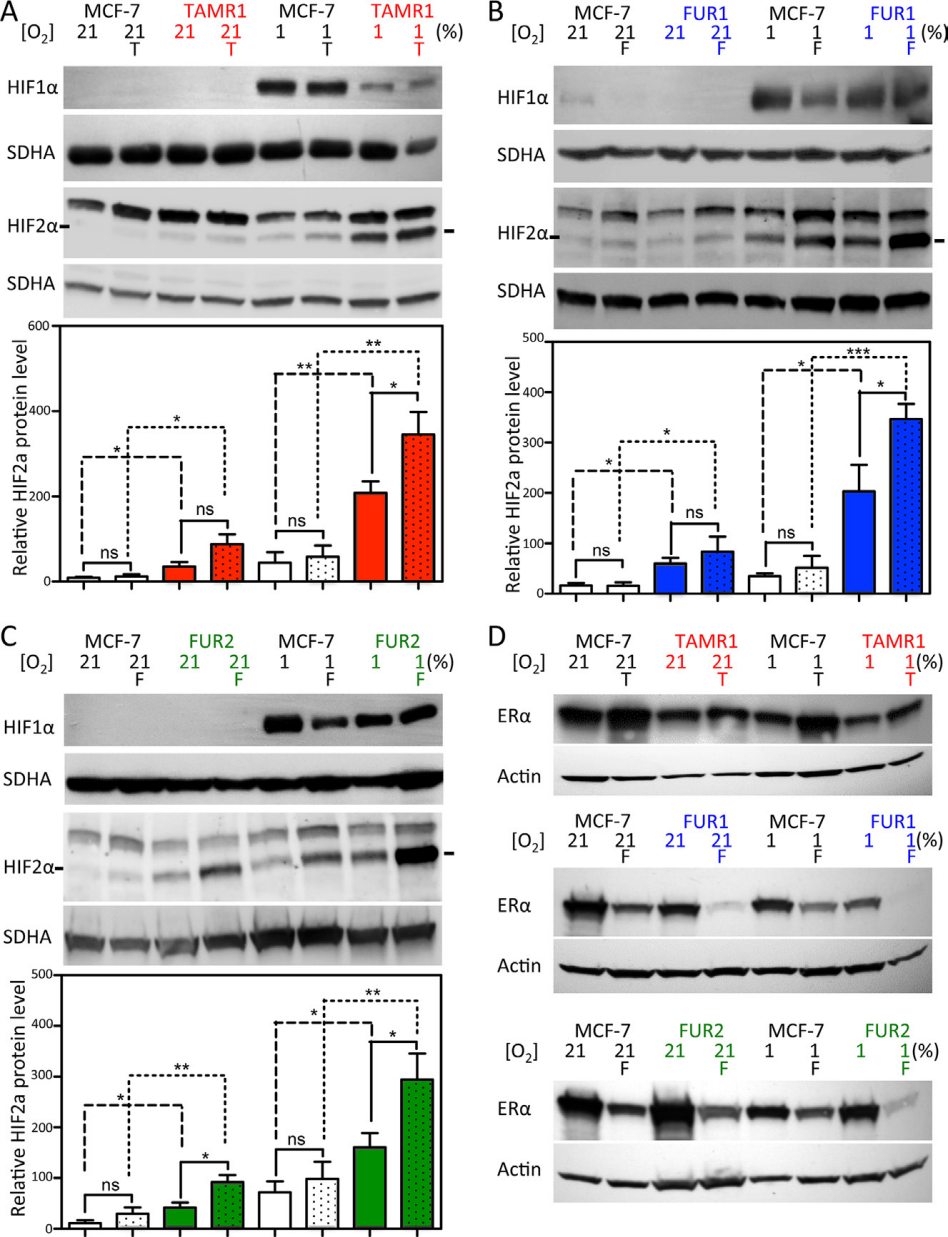
Effect of hypoxia and antiestrogen treatment on HIF1α and HIF2α in MCF-7 and antiestrogen-resistant breast cancer cells (**A–C**) MCF-7-derived cell lines with acquired resistance to tamoxifen (TAMR1) and fulvestrant (FUR1 and FUR2) were exposed to tamoxifen [0.5 μM] (T) or fulvestrant [0.5 μM] (F) under normoxic and hypoxic conditions for 72 h and analyzed for HIF1α and HIF2α protein expression by western blotting. SDHA was used as a loading control. The charts show quantification of the HIF2α levels normalized to SDHA in 3–4 independent experiments. Statistical analyses with one-tailed Student's *t*-test, *p < 0.05, **p < 0.01, ***p < 0.001. (**D**) Western blot analyses of ERα protein levels in whole cell lysates of TAMR1, FUR1, and FUR2 cells compared to MCF-7 parental cells. Cells were harvested 72 h after addition of tamoxifen [0.5 μM] (T) or fulvestrant [0.5 μM] (F) to the growth medium under hypoxic (1%) or normoxic (21%) conditions. Actin was used as a loading control.

We next investigated HIF protein levels in the antiestrogen-resistant cell lines TAMR1, FUR1, and FUR2. All three resistant cell lines expressed HIF1α protein at levels comparable to, or lower than, the parental cell line under normoxic (where HIF1α protein is hardly detectable) and hypoxic conditions at 72 h (Figure 2A–2C, upper panels). HIF2α was detected under normoxic conditions in all three antiestrogen-resistant cell lines, with expression further increasing with hypoxia; however, in parental MCF-7 cells, HIF2α expression was robust mainly under hypoxic conditions (Figure 2A–2C). HIF2α signals were quantified and normalized to SDHA levels (Figure 2A–2C, graphs): both tamoxifen- (TAMR1) and fulvestrant-resistant (FUR1 and FUR2) cells expressed significantly higher levels of HIF2α protein compared to parental cells under normoxic and hypoxic conditions (Figure 2A–2C). Furthermore, HIF2α protein levels increased significantly with drug-exposure in hypoxic antiestrogen-resistant cells, a trend also visible at normoxia (Figure 2A–2C). ERα levels were lower in TAMR1 cells than in MCF-7 parental cells, increased with tamoxifen treatment, and decreased at hypoxia (Figure 2D). FUR1 also expressed less ERα than parental MCF-7 cells and, at hypoxia and in the presence of fulvestrant, ERα levels further decreased and was hardly detectable (Figure 2D). FUR2 cells expressed ERα at levels comparable to MCF-7 cells under control conditions, but in fulvestrant- or hypoxia-treated cells ERα levels were consistently lower (Figure 2D). Since low protein levels of ERα (hypoxia and fulvestrant treatment) and impaired function of ERα (tamoxifen) were associated with high protein levels of HIF2α, we examined the effect of siRNA-mediated knockdown of ERα in these cells. Following reduction of ERα increased protein levels of HIF2α were observed two days after transfection (Supplementary Figure S2).

Treatment of patients with high doses of estrogen has been suggested as a strategy against progressed hormone-responsive breast cancer [20, 21]. Incubation with high concentrations of 17β-estradiol (10 μM) for 72 h (i.e. before decreased cell numbers in treated cultures are seen at 6–7 days) lead to increased HIF2α protein levels in both MCF-7 and antiestrogen-resistant cell lines (Supplementary Figure S2). In the resistant cells, but not in MCF-7, a slight increase in EGFR levels was also seen, which may reflect a stress response, especially in the resistant cell lines, since estrogen induces apoptosis in breast cancer cells adapted to long-term estrogen deprivation [22].

### HIF2A mRNA levels are elevated in antiestrogen resistant cells

**Figure 3:**
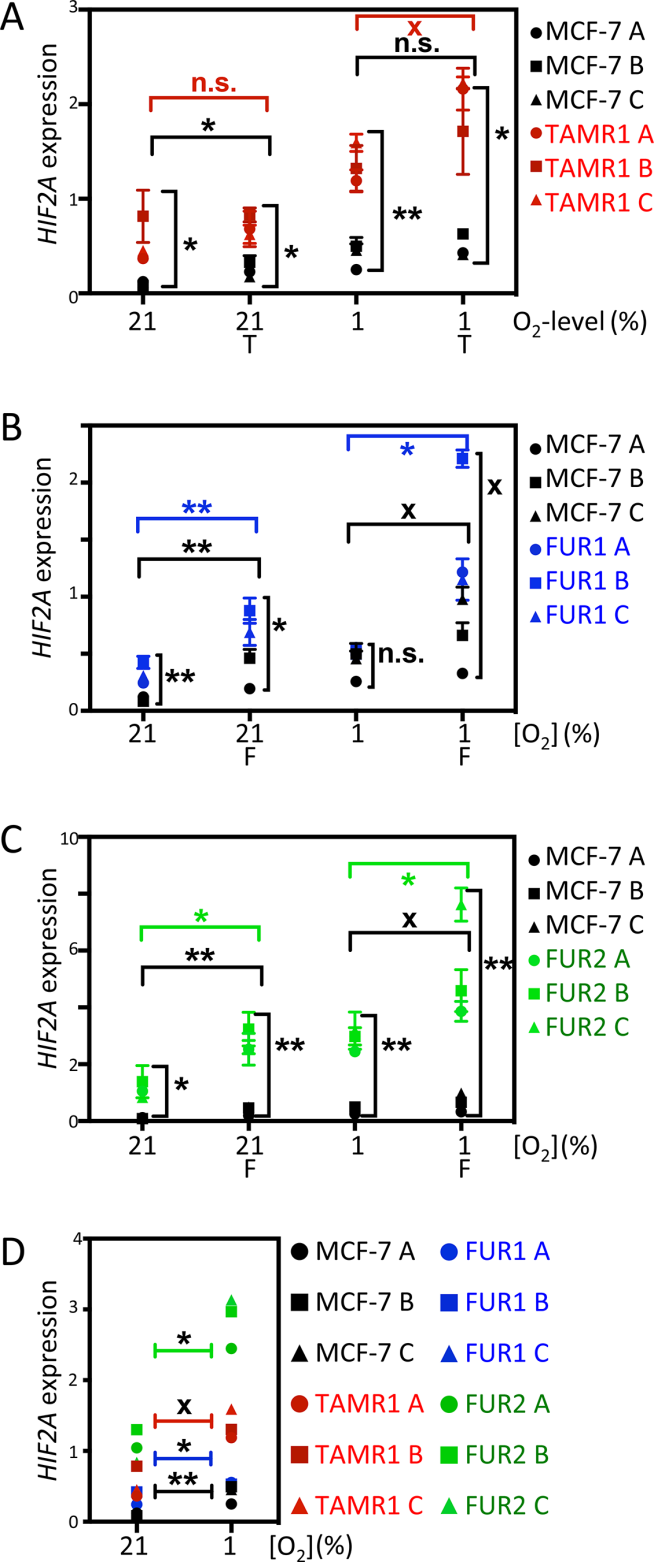
Effect of hypoxia and antiestrogen on HIF2α mRNA expression in MCF-7 and antiestrogen resistant breast cancer cells (**A–C**) Comparison of relative HIF2α mRNA levels in tamoxifen- (TAMR1) and fulvestrant- (FUR1 and FUR2) resistant cells exposed to tamoxifen [0.5 μM] (T) or fulvestrant [0.5 μM] (F) under normoxic and hypoxic conditions for 72 h. HIF2α mRNA levels were measured by qRT-PCR and normalized to the geometric mean of the expression of three reference genes: SDHA, UBC, and YWHAZ. A, B, and C in the chart denote three independent experiments. Statistical analyses with Student's *t*-test, *p < 0.05, **p < 0.01, ***p < 0.001. X acknowledges that the difference between groups was significant * in two out of three independent experiments. (**D**) Direct comparison of HIF2α mRNA levels in the different sub-cell lines at 21% and 1% oxygen. A, B, and C in the diagram denote three independent experiments. Statistical analyses with Student's *t*-test, *p < 0.05, **p < 0.01, ***p < 0.001. X denotes that the difference between the groups was significant * in two out of three independent experiments. HIF2α mRNA as measured in triplicate by qRT-PCR and normalized to the geometric mean of the expression of three reference genes: SDHA, UBC, and YWHAZ.

It is thought that both HIF1α and HIF2α are primarily regulated by protein stabilization in response to low oxygen levels; however, we observed that the protein levels of HIF2α in the developing sympathetic nervous system and in hypoxic neuroblastoma are at least partially regulated transcriptionally [23]. Analysis of *HIF2A (EPAS1)* mRNA expression after 72 h of exposure to tamoxifen and fulvestrant revealed that *HIF2A* mRNA expression was higher in TAMR1, FUR1, and FUR2 cells than in parental MCF-7 cells under normoxic and hypoxic conditions (Figure 3A–3C). Interestingly, *HIF2A* mRNA expression was consistently greater in hypoxic cells than corresponding normoxic cells (Figure 3D). We conclude that increased HIF2α protein expression in breast cancer cells appears to be regulated transcriptionally in response to hypoxia, at least in part. Analysis of published cDNA microarray data [24] revealed 12-fold greater *HIF2A* mRNA expression in four tamoxifen-resistant cell lines (including TAMR1) than the parental cell line (*p* < 0.001), and 26-fold greater mRNA expression in seven fulvestrant-resistant cell lines including FUR1 and FUR2 (*p* < 0.001). In contrast, the *HIF1A* mRNA levels did not vary significantly between the antiestrogen-resistant cells and the parental MCF-7 cells or with exposure to drugs or hypoxic conditions (data not shown), consistent with a post-transcriptional hypoxic induction of HIF1α.

### HIF inhibition restores sensitivity to antiestrogens in resistant cells

We next sought to inhibit HIF2α activity in antiestrogen-resistant cells to test the hypothesis that this would restore antiestrogen sensitivity. As shown above (Figure 1E), HIF2α appears to play a greater role than HIF1α in processes occurring at or after 72 h of hypoxia. Since knock-down experiments using siHIF2A and shHIF2A were unsuccessful over longer periods of antiestrogen exposure (six to nine days), we exploited a commercially available and well-tolerated HIF inhibitor, FM19G11 [25], which inhibits both HIF1 and HIF2 activities and decreases expression of downstream target genes [25].

**Figure 4:**
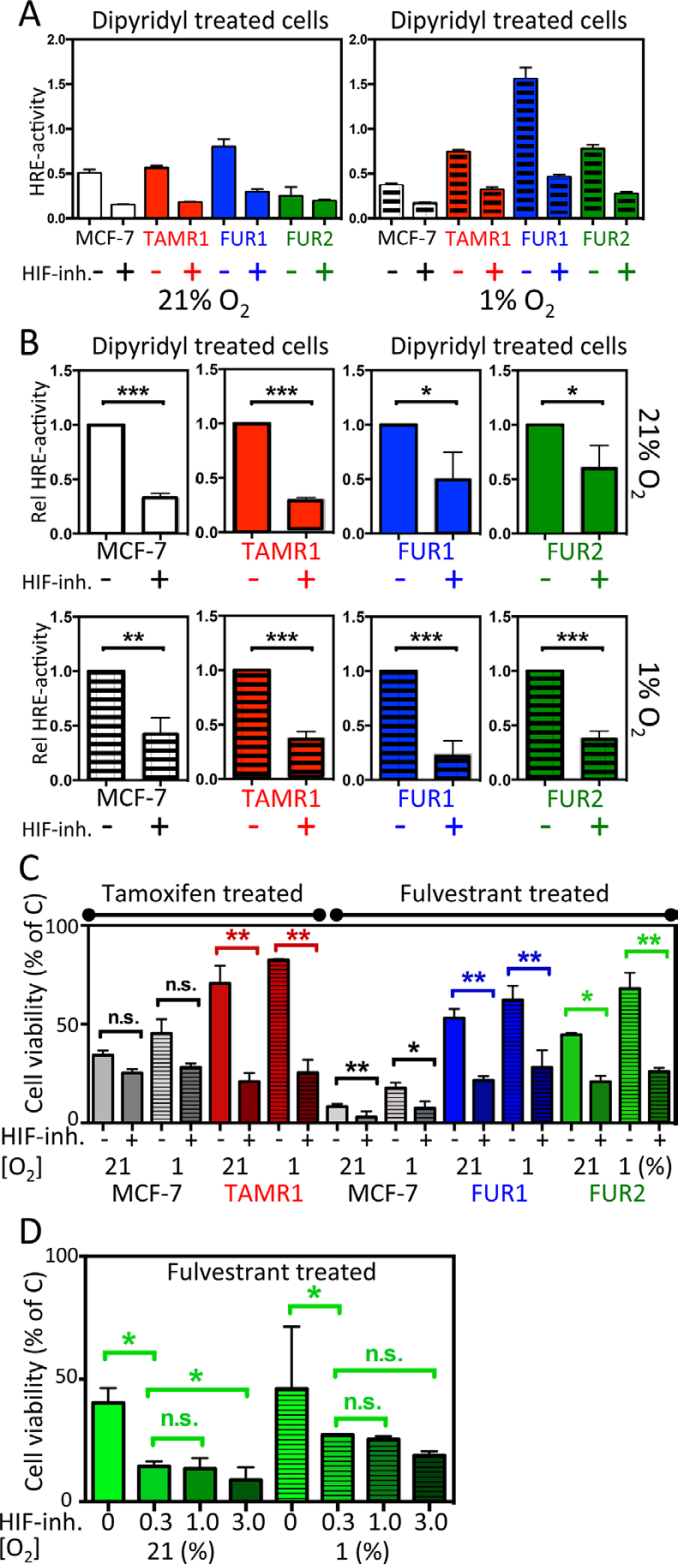
Effect of the HIF-inhibitor FM19G11 on HIF-transcriptional activity and response to antiestrogen treatment in MCF-7 and antiestrogen resistant breast cancer cells (**A–B**) MCF-7, TAMR1, FUR1, and FUR2 cells transiently transfected with the HRE-luciferase reporter and a separate plasmid encoding *Renilla* luciferase were grown under normoxic or hypoxic conditions for 72 h and, over the final 24 h, all samples were exposed to dipyridyl [100 μM] with or without addition of the HIF-inhibitor FM19G11. (A) One representative experiment showing the absolute HRE-luciferase/*Renilla* luciferase activity ratio as the mean and SD of triplicates with and without addition of HIF inhibitor [1 μM]. (B) Statistical analyses of three independent experiments as described in panel A displaying the normalized fraction of HRE activity after addition of the HIF inhibitor [1 μM]. (**C**) Cell survival after exposure to tamoxifen (T) or fulvestrant (F) for six days shown as the percentage of non-drug exposed (C) cells with or without HIF inhibition with FM19G11 [1 μM]. Statistical analyses were with Student's *t*-test, *p < 0.05, **p < 0.01, ***p < 0.001. (**D**) Effect of increasing concentrations of FM19G11 [0, 0.3, 1, and 3 μM] on cell survival in FUR2 cells that had the highest HIF2α levels exposed to fulvestrant.

To ensure efficacy of the HIF-inhibitor, FM19G11, in these cell lines, HRE-luciferase reporter assays were performed to measure HIF-induced transcription at normoxia and hypoxia of all cell lines in the presence and absence of the HIF-inhibitor (Figure 4A and 4B). By inhibiting VHL-dependent proteasomal degradation, DIP treatment accumulates HIFα and activates HIF transcriptional activity, reported via HRE-luciferase activity. Combined exposure to hypoxia further increased HIF transcriptional activity (Figure 4A), which might be due to loss of inhibitory hydroxylation of the HIFα N-terminal transactivating domain by factor inhibiting HIF (FIH-1) when oxygen is available. One μM of FM19G11 led to a several-fold and statistically significant decrease in HRE reporter activity compared to DIP alone in all tested cell lines at 72 h (Figure 4A and 4B). There was a tendency toward decreased HIF2α protein expression in the presence of the HIF inhibitor, (Supplementary Figure S3A).

We next tested the effect of FM19G11 on cell survival in the presence of antiestrogens. In antiestrogen-resistant cells, under normoxic conditions (when HIF2α levels are high and HIF1α levels are low), FM19G11 significantly increased sensitivity to tamoxifen in TAMR1 cells and sensitivity to fulvestrant in FUR1 and FUR2 cells (Figure 4C). A slight increase in sensitivity to tamoxifen and fulvestrant was also observed for MCF-7 parental cells, albeit from very low to even lower viability levels (Figure 4C and Supplementary Figure S4). Increased concentrations of FM19G11 increased the effect of fulvestrant as shown in FUR2 (Figure 4D), the cell line with highest expression level of HIF2α.

### Overexpression of HIF2α increased cell viability in combination with antiestrogen treatment

**Figure 5:**
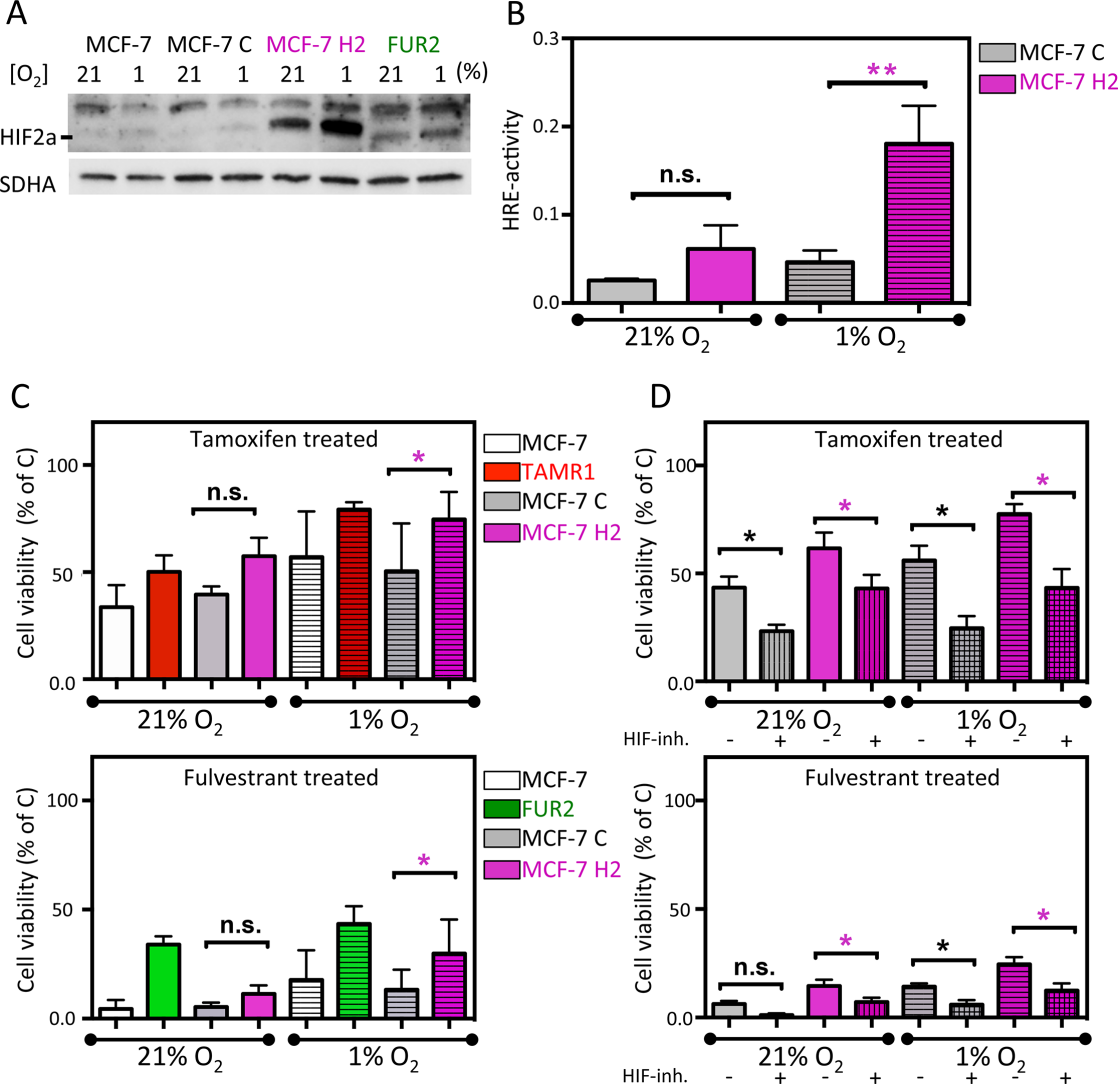
Effect of ectopic expression of stable HIF2α in MCF-7 cells on antiestrogen sensitivity (**A**) Western blot analysis of HIF2α in the parental MCF-7 cells, MCF-7 cells transduced with empty vector (MCF-7C), and with the expression vector for stable HIF2α (MCF-7H2) and, for comparison, FUR2 cells. (**B**) HIF transcriptional activity in the control (MCF-7C) and HIF2A (MCF-7H2)-transduced cells under normoxic and hypoxic conditions were analyzed by HRE-luciferase reporter activity. Data presented is the mean from three independent experiments in triplicate. (**C**) Cell viability of MCF-7 with (MCF-7H2) and without (MCF-7C) overexpression of stable HIF2α and tamoxifen or fulvestrant-resistant cells after 6-day exposure to tamoxifen (upper) or fulvestrant (lower) panel. (**D**) Cell viability of MCF-7 with (MCF-7H2) and without (MCF-7C) overexpression of stable HIF2α and tamoxifen or fulvestrant-resistant cells after 6-day co-exposure to HIF-inhibitor FM19G11 and tamoxifen (upper) or fulvestrant (lower) panel. Three independent experiments of triplicates were performed. Statistical analysis was with Student's *t*-test.

To further examine the link between HIF2α expression and antiestrogen-resistance, an oxygen-stable variant of HIF2α [26] was expressed in parental MCF-7 cells by viral transduction. These cells (MCF-7 H2) expressed high levels of HIF2α protein at normoxia, which further increased with hypoxia (Figure 5A). In agreement, these cells also showed high HRE-luciferase activity, which further increased with hypoxia (Figure 5B). ARNT/HIF1β was essentially uniformly expressed between subclones and conditions (Supplementary Figure S5). Next, we exposed HIF2α-overexpressing MCF-7 cells to antiestrogens and analyzed the percentage of surviving cells after six days at normoxia and hypoxia. The ectopically HIF2α-overexpressing MCF-7 cells were less sensitive to tamoxifen and fulvestrant than parental and control transduced MCF-7 cells (Figure 5C). At hypoxia, the treatment resistance inferred by HIF2α overexpression was even more pronounced, approaching resistance levels seen in TAMR1 and FUR2 cells (Figure 5C). We then co-exposed the virus-transduced cells to antiestrogens and FM19G11 HIF-inhibitor (2 μM) confirming that the HIF2α-induced resistance was counteracted by HIF-inhibition (Figure 5D). In conclusion, overexpression of HIF2α increased the proportion of surviving cells in the presence of antiestrogens compared to control cells, and drug-sensitivity was rescued by HIF-inhibition.

### HIF2α and EGFR protein levels are interdependent in antiestrogen-resistant cells

**Figure 6:**
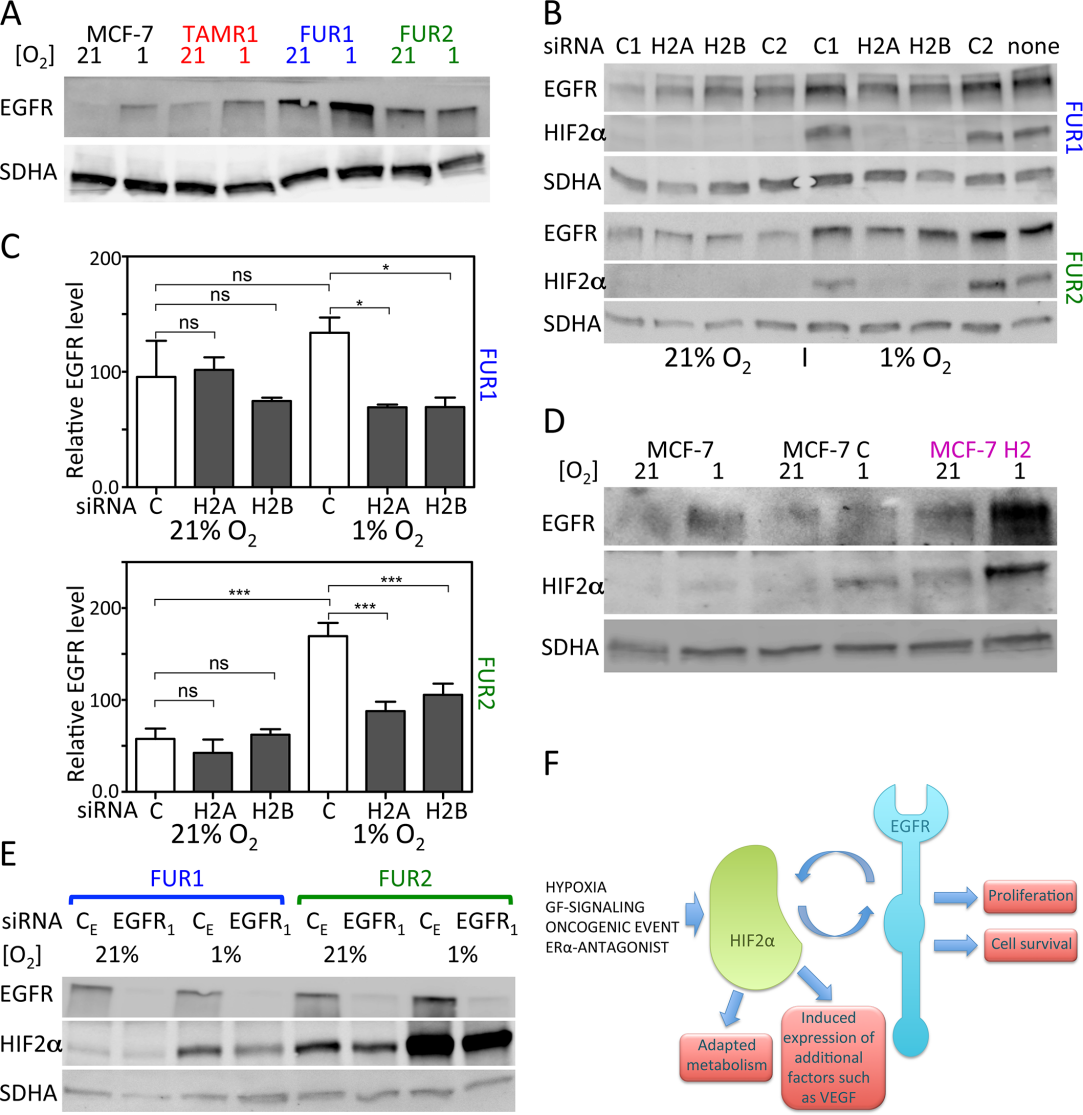
Effect of downregulation of HIF2α on EGFR protein levels and downregulation of EGFR on HIF2α protein levels in MCF-7 and antiestrogen resistant cells (**A**) Protein levels of EGFR in response to 24 h growth under hypoxic conditions in MCF-7, TAMR1, FUR1, and FUR2 cells. SDHA was used as a loading control. One representative experiment out of three independent experiments is shown. (**B**) The effect of downregulation of HIF2α on EGFR protein levels using two different siRNAs (H2A and H2B) was compared to the effect of two control siRNAs (C1 and C2). Samples were harvested 48 h after siRNA transfection and exposure to hypoxia or control conditions for 24 h. SDHA was used as a loading control. (**C**) Quantification of western blots of three independent repetitions of the experiment shown in B. (**D**) The EGFR protein levels in response to HIF2α overexpression by viral transduction (MCF-7 H2) compared to cells transduced with control vector (MCF-7 C). Loading control was SDHA. One representative experiment out of three independent experiments is shown. (**E**) HIF2α protein levels were analyzed in response to EGFR downregulation by siRNA knockdown. One representative experiment out of three independent experiments is shown. (**F**) Our present data suggest that HIF2α induces EGFR, which is a known contributor to resistance to antiestrogen therapy. Furthermore, we show that EGFR can induce HIF2α expression. Thus, EGFR and HIF2α exist with positive crosstalk to induce pro-malignant features including antiestrogen resistance. External factors such as tumor hypoxia and even therapy itself can fuel this interplay, as shown by the increased HIF2α expression in antiestrogen-treated cells.

Since the fulvestrant-resistant cells were reported to express high levels of EGFR that contributes to their resistant phenotype [16], we investigated the effect of hypoxia on EGFR levels in antiestrogen-resistant and parental cells. EGFR expression was higher and increased in response to hypoxia in TAMR1, FUR1 and FUR2 cells (Figure 6A). Since HIF2α is reported to induce EGFR protein levels under hypoxic conditions in renal epithelial cells [14], we tested the effect of HIF2α downregulation by siRNA on EGFR protein levels in antiestrogen-resistant breast cancer cells. HIF2α silencing resulted in significantly lower levels of EGFR in both FUR1 and FUR2 cells at hypoxia (Figure 6B and 6C). Also in TAMR1 cells siHIF2α led to decreased EGFR expression, though initial levels were lower in these cells (data not shown). In agreement with these data, exposure of fulvestrant-resistant cells FUR2 to increasing levels of HIF-inhibitor (FM19G11) lead to increasingly lower EGFR levels (Supplementary Figure S3B). Furthermore, stable ectopic expression of HIF2α in parental MCF-7 cells led to increased levels of EGFR (Figure 6D), confirming HIF2α-driven induction of EGFR.

In addition to hypoxic accumulation, HIF1α and HIF2α can be induced in response to growth factor signaling; therefore, we tested the effect of EGFR downregulation on HIF2α protein expression. siEGFR decreased HIF2α protein levels under normoxia and hypoxia in antiestrogen resistant cells (Figure 6E, Supplementary Figure S6, and data not shown), suggesting that EGFR and HIF2α positively regulate each others expression. Furthermore, inhibiting the activity of EGFR by Gefitinib reduced HIF2α protein levels in the fulvestrant resistant cell line FUR2 (Supplementary Figure S6) especially under normoxic conditions.

## DISCUSSION

The vast majority of women diagnosed with ERα-positive breast cancer receive antiestrogen treatment. Anti-hormonal therapy prevent cancer regrowth and metastasis, but in about 30% of patients receiving adjuvant tamoxifen, and in almost all patients with advanced breast cancer receiving tamoxifen, the cancer becomes resistant [2]. Currently, there is a lack of curative treatment for resistant breast cancer. Overcoming antiestrogen resistance is, therefore, a priority for breast cancer researchers, and a number of factors have been implicated in endocrine resistance including EGFR [16] and HIF-1 [12, 13, 27].

Tumor hypoxia is a well-established negative prognostic factor in cancer, including breast cancer [13, 28, 29]. Here, we show that hypoxia reduces responses to tamoxifen and fulvestrant in ERα-positive cells: MCF-7, CAMA-1, and T47D. The low oxygen conditions induced accumulation of both HIF1α and HIF2α, but after 24 h HIF1α levels were declining whereas HIF2α remained and effects on cell survival after antiestrogen exposure were seen after 6 days of hypoxia and drug exposure. HIF2α protein and mRNA expression were increased in a panel of antiestrogen-resistant cell lines, further establishing a link between hypoxia, HIF2α expression, and treatment resistance. Earlier reports showed that the cell line model of acquired antiestrogen-resistance used in this study, has clinical relevance since protein alterations reported in the resistant cell lines were found also in clinical samples from patients with antiestrogen-resistant breast cancers [30]. Our data demonstrate a direct link between HIF2α and antiestrogen resistance, since ectopic expression of HIF2α induced antiestrogen resistance in otherwise antiestrogen-sensitive breast cancer cells. Furthermore, inhibition of HIF-activity by FM19G11 under conditions where HIF2α is present, and HIF1α levels were not detected, restored antiestrogen sensitivity in the resistant cells, TAMR1, FUR1 and FUR2. Thus, we find HIF2α to be necessary and sufficient for the induction of antiestrogen resistance in the studied cells. Our findings highlight the potential feasibility of targeting HIF2α to overcome antiestrogen resistance. A similar situation is seen in clear cell renal carcinoma, where the negative regulator of HIF1α and HIF2α is frequently lost, and HIF2α is an oncoprotein and HIF1α acts as a tumor suppressor [31]. The transcriptional activity of HIF2α is less strictly regulated by oxygen conditions since factor inhibiting HIF1 (FIH1) has less affinity for HIF2α [32] allowing it to be active at physiological oxygen levels if accumulated as seen in the MCF-7 derived antiestrogen-resistant cell lines here.

Our data suggest that one mechanism whereby HIF2α contributes to the antiestrogen resistance is by increasing the protein levels of EGFR (Figure 6E), which has been shown to play an important role for fulvestrant resistance in cell lines [16] and is inversely linked to antiestrogen-response in clinical samples [11]. EGFR knockdown and inhibition, in turn, demonstrated that EGF-signaling contributes to the high HIF2α levels in the resistant cells (Figure 6 and Supplementary Figure 6). Based on these data, we hypothesize that the collective activities of EGFR and HIF2α lead to therapeutic resistance and potentially other pro-malignant traits such as angiogenesis, proliferation and altered metabolism (Figure 6F). Our data suggest that this “vicious circle” is fueled by hypoxia and even antiestrogen-therapy itself, since tamoxifen, fulvestrant, and ERα downregulation increase HIF2α expression (Figures 2 and 3, and Supplementary Figure S2). Our present data are in line with the earlier publication by Franovic et al. showing that HIF2α confers growth factor-independent proliferation to numerous cancers, including breast cancer, regardless of their tissue of origin or mutational status and that the proliferative effect was exerted via receptor tyrosine kinases, including EGFR [33].

The general view is that HIF1α and HIF2α are mainly regulated post-translationally by oxygen-dependent proteasomal degradation [34, 35]. However, growth factor signaling has been shown to regulate HIF1α at both the transcriptional and translational level [36–41], and HIF2α transcription in hypoxic neuroblastoma cells is regulated by IGF-2 [23, 42]. Here, we show that EGFR knockdown downregulates the high HIF2α levels in antiestrogen-resistant cells (Figure 6E and 6F).

Tamoxifen-resistant cell growth has previously been shown to depend on ERα [30] and recently to be due to crosstalk with the Aurora kinase A [43], which is a determinant of tamoxifen sensitivity through phosphorylation of ERα [44]. Despite that we find ERα protein expression being downregulated by hypoxia, a phenomenon that can be linked to HIF1α [45], ERα signaling persisted under hypoxic conditions (Figure 1C). HIF1α has been reported to promote ligand-independent ERα signaling, presumably, by direct binding of HIF1α to ERα [46]. Given the high homology between HIF1α and HIF2α, HIF2α may also be able to propagate ERα signaling; this requires experimental validation.

As mentioned above, tamoxifen-resistant cells, including TAMR1, depend on ERα-activity also in the presence of tamoxifen [30, 43]. The situation is quite different in fulvestrant treatment and fulvestrant-resistant cells where ERα is lost because of the destabilizing effect of fulvestrant-binding and the resistant cells take advantage of ERα independent mechanisms including activation of HER/EGFR pathways [16]. A similar setting to fulvestrant treatment is the exposure to siERα (Supplementary Figure 2) where we see increased HIF2α levels. The fact that HIF2α infers resistance to both tamoxifen and fulvestrant and that HIF-inhibition/downregulation increases sensitivity to both tamoxifen and fulvestrant indicates that HIF2α confers increased antiestrogen resistance in a general manner.

The frequent presence of tumor hypoxia, HIF1α, and HIF2α in solid tumors, including breast cancer, suggests that these tumor areas may possess inherent antiestrogen resistance that can withstand therapy and contribute to treatment failure. It now appears that antiestrogen treatment itself might exacerbate the resistance phenotype. Our finding that HIF inhibition by FM19G11 restores antiestrogen sensitivity indicates that targeting HIF activity may be a feasible strategy to counteract antiestrogen resistance in clinical practice. However, this requires further investigation in the clinical setting.

## MATERIALS AND METHODS

### Cells and cell culture

The ERα-positive human breast cancer cells MCF-7, CAMA-1, and T47D were obtained from the American Type Culture Collection (ATCC, USA). The MCF-7/S0.5 subline (MCF-7, parental cells) and the antiestrogen resistant strains that were derived from this cell line, MCF-7/TAM^R^-1 (TAMR1), MCF-7/182^R^-6 (FUR1) and MCF-7/164^R^-7 (FUR2) were obtained from Anne Lykkesfeldt, Danish Cancer Society Research Center. The MCF-7 sub-line was established by stepwise reduction of the serum concentration from 5% to 0.5% [17]. The tamoxifen-resistant TAMR-1 cell line and the fulvestrant-resistant cell lines FUR1 and FUR2 were established by long term selection with 1 μM tamoxifen, 0.1 μM ICI 182,780 (fulvestrant) and 0.1 μM ICI 164,384, respectively, as previously described [18, 19]. Due to the long periods of culture, MCF-7 cell line authenticity was tested and positively confirmed (DSMZ, Germany). All cells were grown in standard DMEM/F12 medium (Thermo Fisher Scientific, MA) with FCS (MCF-7, TAMR1, FUR1, and FUR2: 1%; T47D and CAMA-1: 10%, Biosera, MO), penicillin and streptomycin (100 units/ml, Hyclone, GE Healthcare, UT) and insulin (100 units/ml, Actrapid, Novo Nordisk, Denmark). The cells were routinely cultured at 37°C, 5% CO_2_, and air oxygen levels, kept at low passage numbers, and checked for *Mycoplasma* on a monthly basis (results were consistently negative). Antiestrogen-resistant cells were maintained in their respective antiestrogen until 1–2 weeks prior to experimental use. Growth medium with additives was changed every third day. Hypoxic cell culture experiments were performed in a Don Whitley Hypoxystation (Don Whitley Scientific, Shipley, UK) under identical culture conditions except for oxygen. Cells were treated with tamoxifen (4-hydroxytamoxifen; Sigma Aldrich, MO; 0.5 μM) or fulvestrant (ICI 182.780; Tocris Bioscience, UK; 0.5 μM) as indicated.

### Immunoblotting

Whole cell lysates (40–80 μg protein in RIPA buffer with Complete, Roche, Switzerland) were electrophoretically separated (7.5% Mini TGX gel, BioRad Laboratories CA). Protein detection was performed using anti-HIF1α (Becton Dickinson, NJ), anti-HIF1β (Ab126985) anti-HIF2α (Ab199, Abcam, UK), anti-EGFR (DAK-H1-WT, Dako, Denmark), ERα (Cell Signaling Technologies, MA), actin (MP Biomedicals, CA) and SDHA (Ab14715, Abcam).

### RNA isolation, cDNA synthesis, and qRT-PCR

cDNA was generated from purified RNA (QIAshredder, RNeasy, Qiagen, Netherlands, Applied Biosystems). Genomic DNA was removed by RNase-free DNase (Promega, WI). qRT-PCR was performed (7300 RT-PCR, Applied Biosystems) with SYBR Green (Applied Biosystems) using forward and reverse primers for: HIF1α (f-5′TTCCAGTTACGTTCCTTCGATCA3′, r-5′TTTGAGGACT-TGCGCTTTCA3′), HIF2α (f-5′GC TCTCCACGGCCTGATA3′, r-5′TTGTCACAC-CTATGG CATATCACC3′) and EGFR (f-5′CTAATTTGGTGGCTG CCTTTCT3′, r-5′CCCGAGTATCTCAACACTGTCC3′). Gene expression was normalized using the geometric mean of three reference genes: SDHA (f-5′TGGGAACA AGAGGGCATC-TG3′, r 5′CCACCACTGCATCAAATT CATG3′), UBC (f-5′ATTTGGGTCGCGGTT-CTTG3′, r 5′TGCCTTGACATTCTCGATGGT3′), and YWHAZ (f-5′ACTTTTGGTACA-TTGTGTGGCTTCAA3′, r-5′CC GCCAGGACAAACCAGTAT3′).

### Cell counting

Cells were exposed to the specified conditions six days prior to detachment, trypan blue staining, and counting in an automated cell counter according to the manufacturer's protocol (Countess, Life Technologies).

### WST-1 assay

Cells were treated with the specified conditions in 96-well plates for up to nine days. Relative viable cell numbers were measured by WST-1 (Roche) at normoxia after change to normoxic growth medium to minimize the risk that hypoxia would interfere with the detection of metabolically active cells. Optical densities were measured at 450 and 630 nm (Synergy 2, BioTek, VT).

### Luciferase assays

Cells were transfected with HRE-luc or ERE-luc reporter constructs (HRE, Addgene, MA, #26731 deposited by N Chandel; ERE; pGL2 luciferase reporter plasmid with ERα-responsive element) and pRL-SV40 (internal control). For ERE-luc analyses, cells were kept in standard growth medium supplemented with 10 nM estrogen, 1 μM tamoxifen, or both and cultured 72 h in 21% or 1% oxygen. Cell lysates were analyzed using Dual-Luciferase Reporter Assay (Promega) and normalized to internal controls.

### HIF inhibitor

FM19G11 (Calbiochem, Merck Millipore, MA) [25, 47] was diluted in DMSO and used at final concentrations of 0.5–3.0 μM. The corresponding amount of DMSO was added to controls.

### Ectopic expression of HIF2A and gene silencing

Parental MCF-7 cells was transduced with the retroviral vector encoding a stable version of HIF2α HA-HIF2α P531A-pBabe-puro (Addgene; Dr. W.G. Kaelin) [26] or empty pBabe-puro. Transduced cells were selected by puromycin. HIF2α silencing was performed using two custom made siRNAs targeting different HIF2α mRNA regions and two control siRNAs (Life Technologies). EGFR silencing was performed using validated silencer siEGFR (s563 and s564) and controls (Life Technologies). ERα silencing was performed using pre-designed siRNAs (s4823 and s4825) and controls (Life Technologies).

### Statistical analyses

Statistical analyses were conducted in GraphPad Prism 6.0 (GraphPad Software, CA). Student's *t*-test was performed and results are shown as mean +/– 1SD with *p*-values: *p < 0.05, **p < 0.01, ***p < 0.001.

## SUPPLEMENTARY MATERIALS FIGURES


