# Supplementary material for: HIF2α contributes to antiestrogen resistance via positive bilateral crosstalk with EGFR in breast cancer cells
Source: Oncotarget. 2016 Feb 3;7(10):11238–50. doi: 10.18632/oncotarget.7167 (PMC4905469; doi:10.18632/oncotarget.7167)
Supplement: Supplementary file 1 [file oncotarget-07-11238-s001.pdf]

HIF2α contributes to antiestrogen resistance via positive bilateral crosstalk with EGFR in breast cancer cells

Supplementary Materials

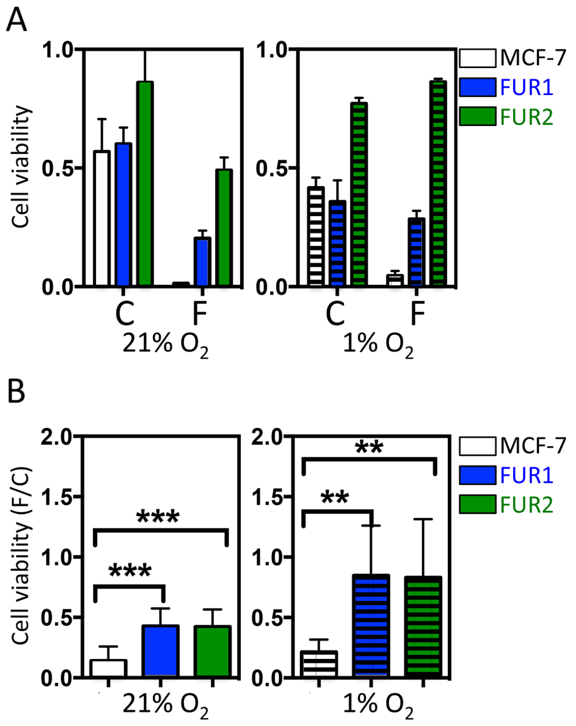

Supplementary Figure S1: Fulvestrant sensitivity in FUR1 and FUR2 cells compared to parental MCF-7 cells. (**A**) Absolute absorbance of a representative WST-1 analysis of FUR1 and FUR2 compared to MCF-7 parental cells under normoxic (21%) and hypoxic (1%) conditions with or without addition of fulvestrant [0.5 μM] (F) to the growth medium after nine days of culture. Bars show mean with SD of triplicates. (**B**) The relative WST-1 absorbances (ratio between fulvestrant-treated/non-treated cells) are displayed. Bars show the mean of nine independent experiments with SD. WST-1 in fresh cell culture medium was added to the cells after returning the cells to normoxic conditions. Statistical analyses were with Student's *t*-test, $*p < 0.05$, $**p < 0.01$, $***p < 0.001$.

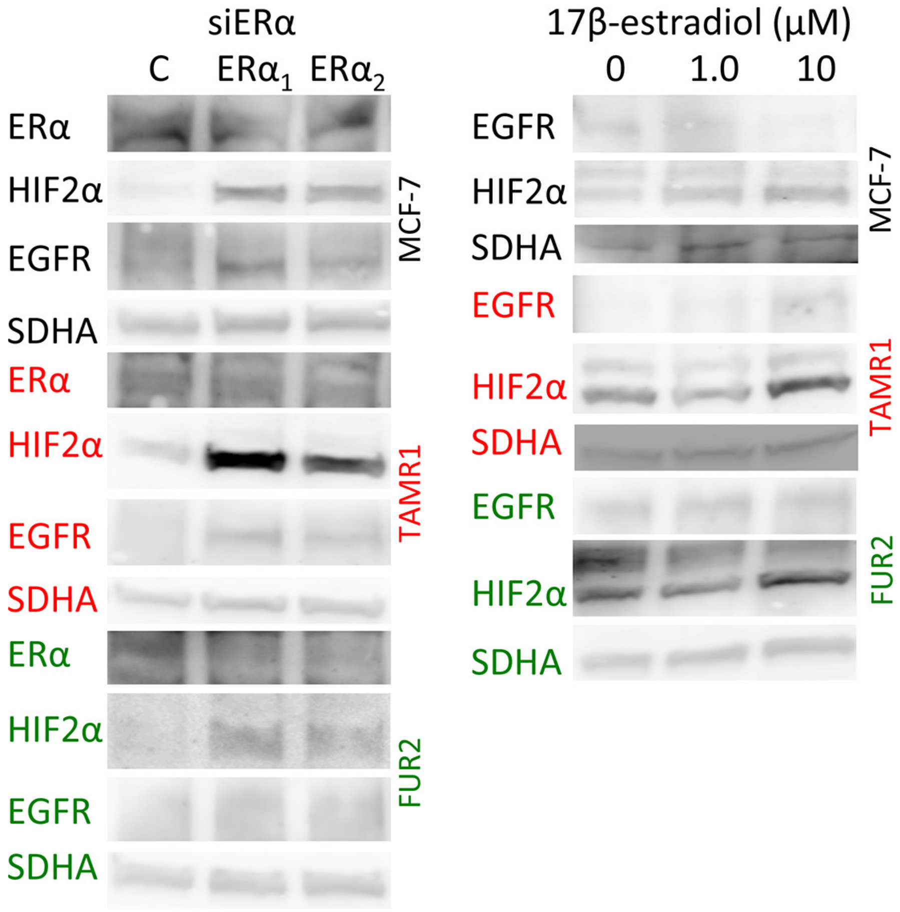

Supplementary Figure S2: Effects of therapeutic concentrations of 17β-estradiol and siRNA-mediated knockdown of ERα in MCF-7, TAMR1 and FUR2 cells. Western blot analyses of MCF-7, TAMR1 and FUR2 cells exposed to siRNA against ERα (left) or high-dose 17β-estradiol (1.0 and 10 µM) (right).

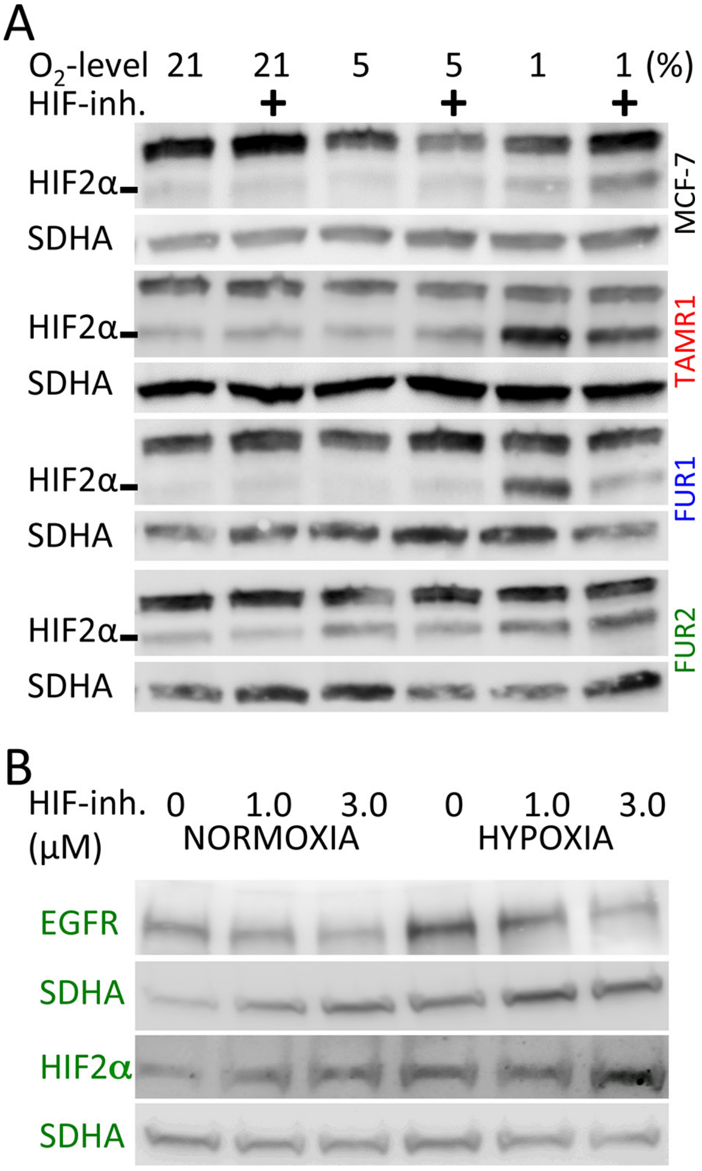

Supplementary Figure S3: Effect of the HIF-inhibitor FM19G11 on HIF2α and EGFR levels. Western blot analyses of HIF2α levels in MCF-7, TAMR1, FUR1, and FUR2 cells in the presence and absence of the HIF-inhibitor FM19G11 (**A**). Analysis of EGFR expression in fulvestrant-resistant cells (FUR2) in response to HIF-inhibitor exposure (**B**).

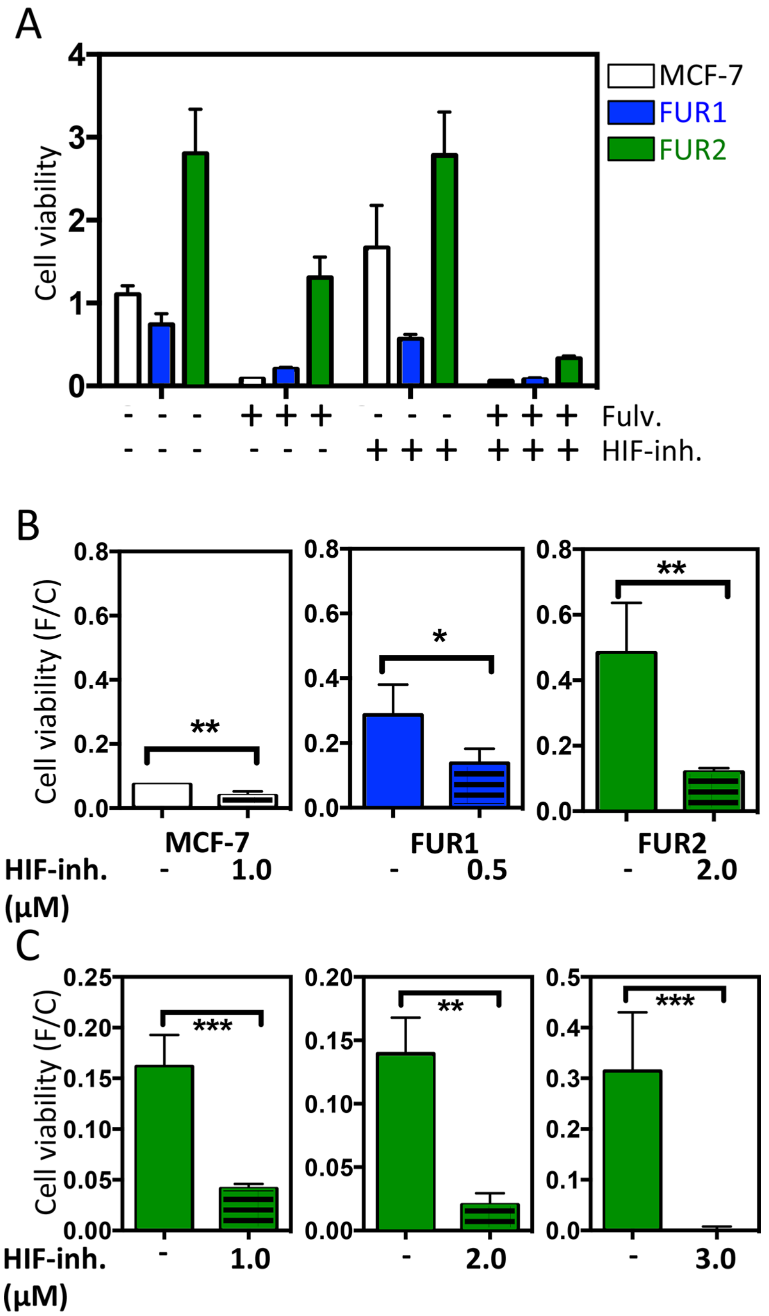

Supplementary Figure S4: Effect of HIF-inhibitor FM19G11 on antiestrogen sensitivity. (**A**) The relative amount of viable cells (absolute absorbance in WST-1 assay) after nine days exposure to growth medium with or without fulvestrant [0.5 µM] (F) and with or without addition of optimized levels of HIF-inhibitor [FUR1 0.5 µM, MCF-7 1.0 µM, and FUR2 2 µM]. (**B**) The relative viability in fulvestrant exposed/control cells from the same representative experiment as shown in (A) Statistical analysis of triplicates with Student's *t*-test, *$p < 0.05$, **$p < 0.01$, ***$p < 0.001$. (**C**) Increasing effect on number of viable FUR2 cells, with the highest HIF2α levels of the tested cell lines, exposed to fulvestrant [0.5 µM] with increasing concentrations of the HIF inhibitor. In all cases, data from normoxic conditions are displayed i.e., presence of HIF2α in resistant cells but very low to non-detectable levels of HIF1α.

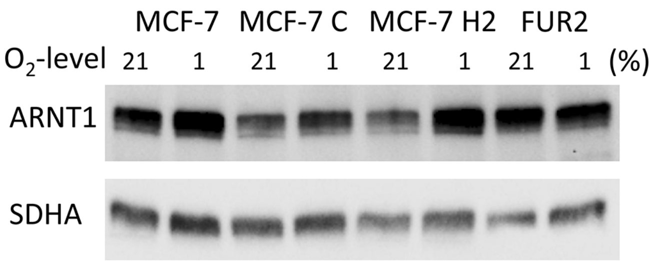

Supplementary Figure S5: Expression of HIF1β/ARNT1 in MCF-7 with and without ectopic expression of HIF2α. Western blot analysis of HIF1β/ ARNT1 expression under normoxic and hypoxic conditions in parental MCF-7 cells, control vector (**C**), HIF2A vector (**H**) transduced, and FUR2 cells 48 h post transfection and 24 h at hypoxia or control conditions.

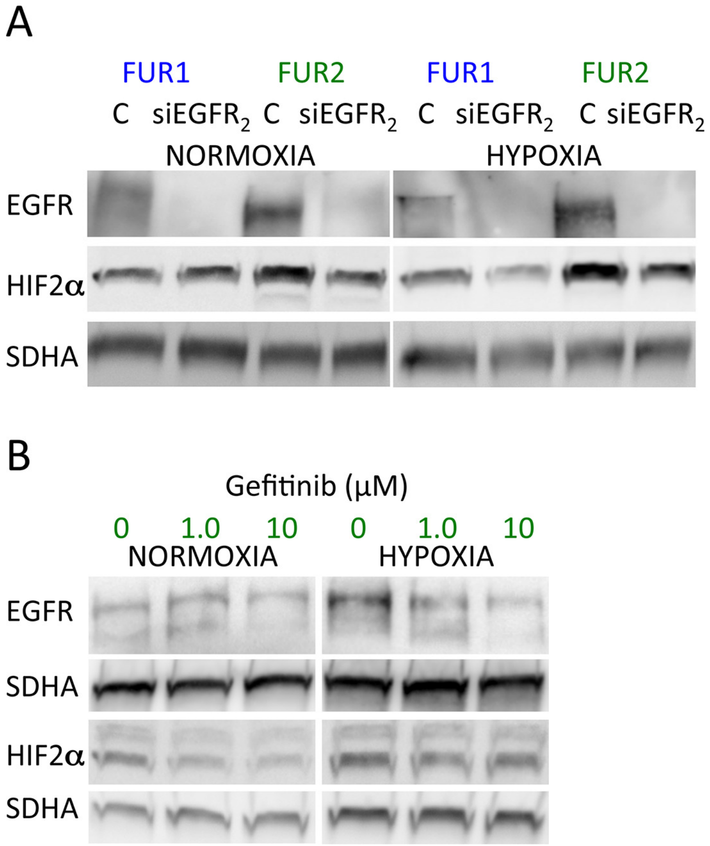

Supplementary Figure S6: Effect of siRNA-mediated knockdown of EGFR and inhibition of EGFR by Gefitinib under normoxia and hypoxia. Western blot analyses of FUR1 and FUR2 cells transfected with siRNA against EGFR (siEGFR$_2$) or control (C) (**A**) and the EGFR inhibitor Gefitinib (1.0 and 10 µM) (**B**).
